# Energy Consumption in Capacitive Deionization for Desalination: A Review

**DOI:** 10.3390/ijerph191710599

**Published:** 2022-08-25

**Authors:** Yuxin Jiang, Linfeng Jin, Dun Wei, Sikpaam Issaka Alhassan, Haiying Wang, Liyuan Chai

**Affiliations:** 1 School of Metallurgy and Environment, Central South University, Changsha 410083, China; 2 Chemical and Environmental Engineering Department, College of Engineering, University of Arizona, Tucson, AZ 85721, USA; 3 Chinese National Engineering Research Center for Control and Treatment of Heavy Metal Pollution, Changsha 410083, China; 4 Water Pollution Control Technology Key Lab of Hunan Province, Changsha 410083, China

**Keywords:** energy consumption, capacitive deionization, desalination, charge efficiency, energy recovery

## Abstract

Capacitive deionization (CDI) is an emerging eco-friendly desalination technology with mild operation conditions. However, the energy consumption of CDI has not yet been comprehensively summarized, which is closely related to the economic cost. Hence, this study aims to review the energy consumption performances and mechanisms in the literature of CDI, and to reveal a future direction for optimizing the consumed energy. The energy consumption of CDI could be influenced by a variety of internal and external factors. Ion-exchange membrane incorporation, flow-by configuration, constant current charging mode, lower electric field intensity and flowrate, electrode material with a semi-selective surface or high wettability, and redox electrolyte are the preferred elements for low energy consumption. In addition, the consumed energy in CDI could be reduced to be even lower by energy regeneration. By combining the favorable factors, the optimization of energy consumption (down to 0.0089 Wh·g_NaCl_^−1^) could be achieved. As redox flow desalination has the benefits of a high energy efficiency and long lifespan (~20,000 cycles), together with the incorporation of energy recovery (over 80%), a robust future tendency of energy-efficient CDI desalination is expected.

## 1. Introduction

Capacitive deionization (CDI) is the process of removing salt ions by electrochemical adsorption [1,2,3,4,5,6,7,8], which is a promising technology for desalination without intensive working conditions and secondary pollution [9,10,11]. Moreover, CDI has already been put into practical use for industrial desalination by commercial companies such as EST Water and Technologies (Changzhou, China) and Atlantis Technologies (Dana Point, CA, USA) [12]. Numerous studies about CDI have been carried out to upgrade the desalination efficiency of CDI [13,14,15,16,17,18,19,20,21,22,23]. However, energy consumption, as a key parameter that directly determines the operation cost, has not received sufficient attention and has played a supporting role in addition to the desalination capacity in the research on CDI for a long period.

The energy consumption of CDI is influenced by various factors. The cell framework, electrode status, electric field mode, and electrode material property are the critical ones. This review summarizes the impacts of these elements into different categories, and the particular mechanisms that influence CDI energy consumption are analyzed. The energy consumption performance has been improved to be as low as 0.01 Wh·g_NaCl_^−1^ by optimizing the relevant factors, which is over 99% lower than that of the traditional CDI process, indicating obvious progress in the field of CDI energy efficiency. In addition, deionization desalination is a process of electrical charging equivalent to energy storage, and even lower energy consumption could be achieved by regenerating the consumed energy. As an example, for comparison, the total energy consumption (energy consumed deducted energy regenerated) of CDI with a feed solution of around 30,000 ppm was reduced to 0.0089 Wh·g_NaCl_^−1^ with energy recovery, nearly 93.3% lower than that of reverse osmosis (RO) technology with the same feed salinity. Thus, there is no theoretical ceiling for the optimization of energy savings for CDI. Moreover, redox flow desalination with energy recovery could combine the advantages of low energy consumption, high desalination efficiency, fast ion diffusion, and excellent cyclic ability, revealing a robust future for research on the low-cost desalination technique. From an electrochemical and dynamical point of view, energy consumption in CDI is related to charge transfer and ion diffusion. With this consideration, the relevant details in the publications were gathered and sorted into varied factors that impact CDI energy consumption. This study aimed to collect and summarize the scrappy information about the energy consumption of CDI in the literature to provide a comprehensive view of the state-of-the-art of the energy consumption of CDI and its mechanism, and further provide insight into future trends in the reduction of CDI energy consumption at the same time.

## 2. The Impacts of the Device Configuration on the Energy Consumption of CDI

### 2.1. Membrane CDI

In traditional CDI systems, the electrodes attract oppositely charged ions and expel similarly charged ions during desalination, thus wasting energy, resulting in a low charge efficiency and desalination capacity, which is called the “co-ion effect” [24,25,26,27]. The incorporation of ion-exchange membranes is a feasible solution to this. When the CDI cell is being charged, the membrane blocks oppositely charged ions from leaving the electrode, resulting in a higher desalination performance and lower energy consumption.

**Figure 1:**
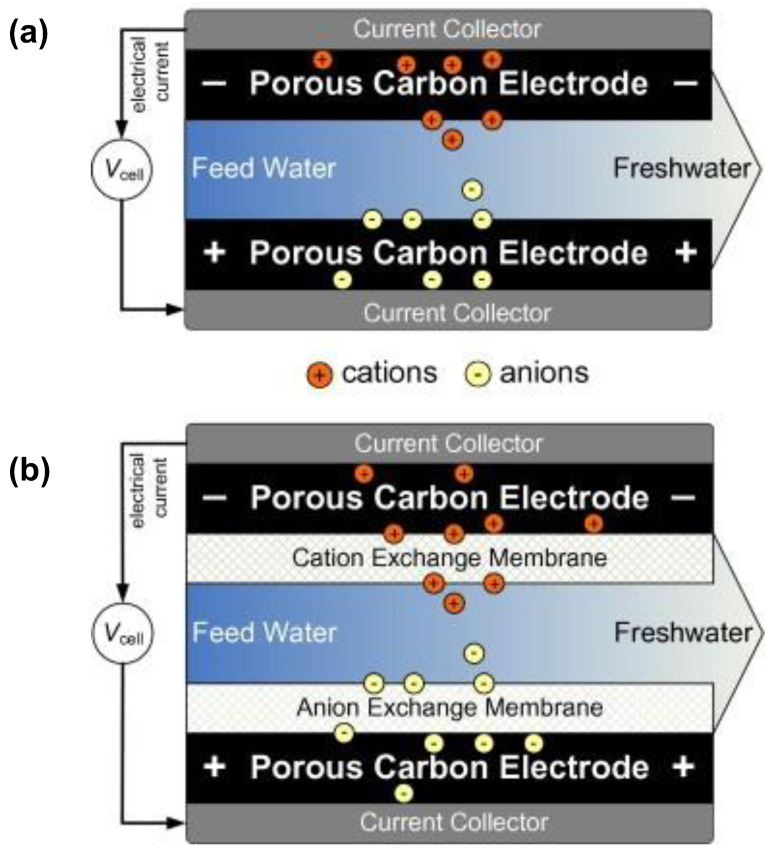
Comparison of (**a**) CDI cell and (**b**) membrane CDI cell with carbon electrodes. Reproduced with permission from [3]. Copyright 2013 Elsevier.

Capacitive deionization with the incorporation of an ion-exchange membrane is named the “MCDI (membrane CDI)” as shown in Figure 1 [3]. Lee et al. [28] employed activated carbon cloth as the electrode material with ion-exchange membranes to hinder the desorption of co-ions. A salt removal efficiency increase of 19% was observed, representing an innovative study of MCDI. Kim et al. [29] demonstrated that salt ions could be retained in the pores of carbon electrodes in traditional CDI. Meanwhile, a similar phenomenon was not observed in MCDI, with 32.8–55.9% higher desalination capacities and higher charge efficiencies (83.9–91.3% versus 35.5–43.1% in a 200-ppm salt solution). In the CDI process, under constant voltage mode, energy consumption is inversely proportional to the charge efficiency as illustrated in Equation (1) (detailed derivation of the equation and the calculation of the other parameters are presented in the Supplementary Materials):

(1) $$W\Lambda =\frac{UF}{3600\times M_{NaCl}}$$

In the above equation, *W* (Wh·g_NaCl_^−1^) stands for the specific energy consumption, *Λ* is the charge efficiency, *U* (V) refers to the applied voltage in constant voltage mode, *F* (96,485 C·mol^−1^) demonstrates the Faraday constant, and *M_NaCl_* (g·mol^−1^) is the molar mass of NaCl. As such, lower energy consumption of MCDI could be inferred.

Zhao et al. [30] utilized graphite cloths as CDI and MCDI electrodes and carried out an overall comparison of the energy consumption. In a salt solution of 400 ppm, the MCDI cell showed higher desalination capacities and lower energy consumption from 1.5 to 3.5 V compared with CDI. With the ion-exchange membrane, the average charge efficiency increased from 16.33% to 70.15%. A deionization capacity of 5.62 mg·g^−^^1^ at 3.0 V was obtained for MCDI. The salt removal capacity and energy consumption were 29.79% and nearly 60% lower than those of CDI, respectively. At 3.5 V, the energy consumption of MCDI was 1.54 Wh·g_NaCl_^−1^, 79.13% lower than that of CDI. Additionally, ion-exchange membranes can protect the electrode materials from side reactions, which is also beneficial to energy savings [31]. The comparison of CDI and MCDI is further shown in Table S1.

### 2.2. Flow Electrode CDI

Flow electrode CDI (FCDI) is a CDI process, with electrode particles suspended in aqueous media [32,33,34]. Ion-exchange membranes are used in FCDI to separate saline water and flow electrodes. Owing to the use of ion-exchange membranes, the energy consumption of FCDI is also less than that of traditional CDI [35].

Ma et al. [36] discovered that the energy consumption of FCDI could be lowered by increasing the content of the flow electrode material. When the carbon content increased from 0% to 10% in the 2000-ppm NaCl electrolyte, the energy consumption decreased by approximately 40% in the FCDI device at the initial salinity of 2000 ppm, which was likely due to the elevated charge transfer being enhanced by the suspended carbon loading.

### 2.3. Flow-By and Flow-Through Cells

**Figure 2:**
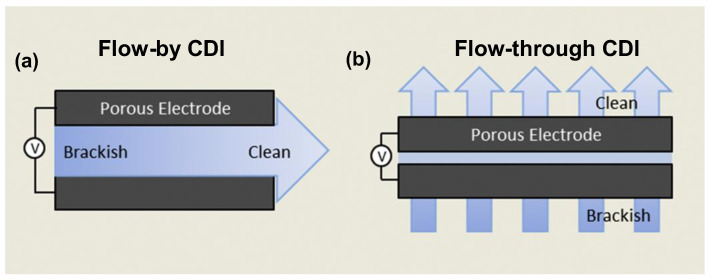
Comparison of (**a**) flow-by CDI cell and (**b**) flow-through CDI cell with porous electrodes. Reproduced with permission from [3]. Copyright 2013 Elsevier.

Few researchers have studied the influences of flow directions on energy consumption. As such, CDI systems can be basically divided into two categories without the consideration of membranes: flow-by and flow-through cells, as presented in Figure 2 [37]. The flow direction of brackish water is parallel to the electrode plates in flow-by CDI but vertical to the electrode plates in flow-through CDI, in which the saline water can penetrate the electrode plates.

Remillard et al. [37] demonstrated that at 1.2 V, in a 292-ppm NaCl solution, flow-by CDI with carbon cloth electrodes showed a desalination capacity of 6.4 mg·g^−1^, which was 48% higher than that of flow-through CDI. The charge efficiency was over 100% higher in flow-by CDI, implying lower energy consumption, which was likely due to the lower internal contact resistance and fewer side reactions. In addition, flow-by CDI suffers from lower feed pressure [38] and shows better stability [31]. Flow-by CDI tends to have a better energy consumption performance than CDI.

Briefly, the suppression of the co-ion effect by an ion-exchange membrane greatly reduces the energy consumption of CDI, and flow-by CDI appears to have more convenient energy consumption than that of flow-through CDI; however, more research work in this area needs to be carried out.

## 3. The Impacts of the Electrical Field and Feed Solution Properties on the Energy Consumption of CDI

For a CDI desalination process, the energy consumption is not only influenced by the internal factors mentioned above but also by the experimental conditions such as the charging mode, voltage or current density, salinity, and flow rate.

### 3.1. Charging Modes

There are mainly two types of charging modes in CDI technology: constant voltage (CV) and constant current (CC). According to the current study, the CC mode is more energy-efficient than the CV mode in CDI despite a lower desalination capacity [39].

By reaching a certain amount of charge applied or salt removal, Kang et al. [40] found that the CC mode showed an energy consumption that was 26–30% lower than that of the CV mode and a lower salt removal performance due to the lower cell voltage during charging. Choi [41] reported that the energy consumption of the CV mode employing activated carbon electrodes was 0.48 Wh·g_NaCl_^−1^ in a 10 mM salt solution, about 60% higher than that of CC, together with a 12% higher desalination capacity. The reason why CC prevails over CV in the energy aspect is likely its fewer side energy storage reactions as reported by Wang et al. [42]. Moreover, in Equation (1), it is also noticeable that for a certain applied voltage, the energy consumption is restricted by the charge efficiency. For example, the energy consumption of the CV mode at 1.4 V is calculated to be 0.64 Wh·g_NaCl_^−1^ when the charge efficiency reaches 100% while Huang et al. [43] achieved an energy consumption lower than 0.18 Wh·g_NaCl_^−1^ with a voltage window of 0–1.4 V during CC CDI.

### 3.2. Electric Field Intensity

Basically, the stronger the electric field is, the higher the energy consumption and desalination performance in the CDI process. There is a trade-off between salt removal and the energy efficiency from the viewpoint of thermodynamics [44]. A higher salt removal performance signifies a higher Gibbs free energy change in the separation reaction and a larger inner resistance of the salt solution caused by the lower transient salinity during the desalination process [45].

Most CDI performances are carried out with the CV or CC mode. Thus, the voltage and current density are the two critical metrics representing the electric intensity in CDI. A higher voltage within the window of water electrolysis could lead to a higher charge efficiency due to the suppression of the co-ion effect [3]. However, according to Equation (1), the specific energy consumption is proportional to the applied voltage under the CV mode and side reactions are more likely to occur as the voltage increases. A higher voltage generally causes higher energy consumption. During the CV CDI research conducted by Chen et al. [46], the energy consumption and charge efficiency increased simultaneously when the voltage increased. Halabaso et al. [47] used manganese-vanadate-decorated reduced graphene oxide as electrodes in a symmetric CDI cell, the energy consumption of which increased by nearly 3 times as the applied voltage increased from 0.8 to 1.4 V, indicating the drastic impact of the electric field intensity on the energy consumption of CDI. Some researchers have even used narrow voltage windows to reduce the specific energy consumption during CC charging, which is further discussed in Section 4.2.

A higher current density could also lead to higher energy consumption under the CC mode due to larger polarization of the circuit [48]. As reported by Xing et al. [49], the CC mode was applied to a hybrid CDI (HCDI) system with a sodium-ion intercalation electrode Na_3_(VO)_2_(PO_4_)_2_/reduced graphene oxide and a capacitive electrode activated carbon. The energy cost increased from 0.35 to 0.40 Wh·g_NaCl_^−1^ when the current density increased from 25 to 100 mA·g^−1^. Wang et al. [45] discovered that the charge efficiency decreased while the energy consumption increased as the current density increased during CDI with the CC mode. Reale et al. [50] obtained an increase in the energy spent (from 0.033 to 0.15 Wh·g_NaCl_^−1^) as the constant current density increased from 1 to 8 mA·cm^−2^ in a CDI system with nickel hexacyanoferrate electrodes with an initial salinity of 100 mM.

### 3.3. Flow Rate

Generally, the flow rate has a negative correlation with the energy efficiency in CDI, which is another reflection of the trade-off phenomena of desalination productivity and energy consumption in the CDI process [51]. Fast kinetics can lead to a low energy efficiency.

Zhao et al. [52] examined the influence of the flow rate on CDI energy consumption with various initial salt concentrations in a CDI cell with porous carbon electrodes. The experimental data showed that energy consumption was higher with a higher flow rate. For example, with a water recovery rate of 50% and an initial salinity of 40 mM, energy consumption with a flow rate of 15 mL·min^−1^ was nearly half of that with 30 mL·min^−1^ and about one-third of that with 60 mL·min^−1^, which was the result of the lower internal resistance caused by the lower flow rate. Qin et al. [53] performed energy consumption tests with a CC CDI mode. As the water flux rose from 5 to 10 L·m^−2^·h^−1^, the energy consumption increased by 43.9%, and when the water flux was moderated to 20 L·m^−2^·h^−1^, the energy consumption was further increased by 32.3%.

### 3.4. Initial Salt Concentration

Interestingly, from the comparison and analysis above, it seems that external factors beneficial to a higher desalination performance could result in higher energy consumption performances. Many studies have indicated that high initial salinities lead to higher energy consumptions. Patel et al. [54] increased the initial salinity of CDI from 3000 to 10,000 ppm and the energy consumption increased by nearly 10 times. Nevertheless, the influence of the initial salinity on CDI energy consumption might be partly different. In a study reported by Zhao et al. [55], it was demonstrated that an initial salt concentration that is too low could also undermine the energy consumption performance, owing to the large charge transfer resistance of the dilute electrolyte, which reduces the charge efficiency.

## 4. The Impacts of the Electrode Materials on the Energy Consumption of CDI

### 4.1. Capacitive Materials

For many years, carbon-based capacitive materials such as activated carbon (AC), carbon nanotubes (CNTs), graphite, graphene, graphene oxide (GO), reduced graphene oxide (rGO), carbon aerogels, etc. have been used as CDI electrode materials [56,57,58,59,60,61,62]. Compared with newly developed electrode materials, capacitive materials show relatively high energy consumption owing to the ion storage mechanism of electrical double layers (EDLs), which leads to non-selectivity of the electrode surfaces, favoring the co-ion effect.

Han et al. [63] set a typical example by utilizing porous activated carbon cloth electrodes in a symmetric CDI system without membranes, delivering an energy consumption of 0.53 Wh·g_NaCl_^−1^ with a constant current density of 0.52 mA·cm^−2^ and an initial salinity of 37.2 mM. Graphene and carbon aerogel are advanced capacitive materials with high conductivities and large surface areas [64]. Kang et al. [65] employed three-dimensional graphene (surface area: 1492.8 m^2^·g^−1^) with ion diffusion channels of mesopores and micropores as electrodes in a symmetric CDI device, the consumed energy of which was 0.16 Wh·g_NaCl_^−1^ at 1.2 V. Xu et al. [66] utilized carbon aerogel electrodes in a CDI test with the CC mode and obtained a relatively low energy cost of 0.21 Wh·g_NaCl_^−1^. Apart from direct usage as electrodes, capacitive materials can also be used to improve energy consumption performances by acting as conductive additives of electrode materials. Sriramulu et al. [67] constructed an HCDI cell with a sodium-intercalation anode and an AC cathode. Due to the combination of rGO in both electrodes, the energy consumption was lowered from 0.64 to 0.39 Wh·g_NaCl_^−1^ at a constant current density of 0.16 mA·cm^−2^.

### 4.2. Intercalation Materials

Since Lee et al. [68] employed Na_0.44_MnO_2_ as the cathode in a hybrid CDI cell, there have been a plethora of studies using intercalation materials as electrodes. Battery materials in solid forms are used as CDI materials with high desalination efficiencies for their high electrochemical capacities, which are often incorporated with carbon to enhance the charge transfer [69,70]. On the other hand, the use of faradaic materials as CDI electrodes could also lower the energy consumption of CDI.

The co-ion effect can be suppressed by intercalation materials by providing perm-selective surfaces to certain salt ions, thus enhancing the ratio between the amount of adsorbed ions and desorbed ions, which leads to a higher charge efficiency and lower energy consumption [71]. Additionally, the higher desalination capacity that results from the ion storage behavior in the crystal structure is also beneficial to a lower specific energy consumption based on the per unit weight of salt removal.

As mentioned previously, the CC mode outperforms the CV mode in CDI energy consumption performance. Some low energy costs were achieved by researchers using battery electrodes with the CC mode. Sodium-intercalation and chloride-intercalation battery materials are the major candidates employed as battery electrodes in CDI cells with low energy consumption.

Vafakhah et al. [72] employed a sodium-intercalation material Fe_4_[Fe(CN)_6_]_3_/rGO as the cathode and rGO as the anode in a hybrid CDI system with an initial salt concentration of 2500 ppm. Within the voltage window of −0.2–1.4 V, a high desalination capacity of 80 mg·g^−1^ and a low energy consumption of 0.23 Wh·g_NaCl_^−1^ were achieved simultaneously with a current density of 0.1 A·g^−1^. Liu et al. [73] used the chloride battery material Bi/C and BiOCl/C as the anode and cathode in a CDI system with an initial salinity of 20 mM, and a low energy consumption of 0.23 Wh·g_NaCl_^−1^ was obtained.

Ultralow energy consumption can be achieved by applying a narrow voltage window to a certain electrode pair in CDI. For example, Srimuk et al. [74] used the highly reversible electrode pair Ag and AgCl in a CDI system with a cation-exchange membrane as the separator. When a voltage was charged, the AgCl cathode released chloride ions into the catholyte NaCl solution, attracting sodium ions from the brackish solution on the other side of the membrane, and the chloride ions in the salt solution were adsorbed by the Ag anode at the same time. The system was mainly related to the intercalation/deintercalation behaviors of chloride ions. Therefore, the cell was denoted as a chloride ion desalination (CID) device. The CID cell was used with an initial salinity of 600 mM and the constant current method, with a current density of 0.1 A·g^−1^ and a voltage window of −0.1–0.1 V. An ultralow energy consumption of 0.059 Wh·g_NaCl_^−1^ was achieved with a high salt removal capacity of 115 mg·g^−1^.

**Figure 3:**
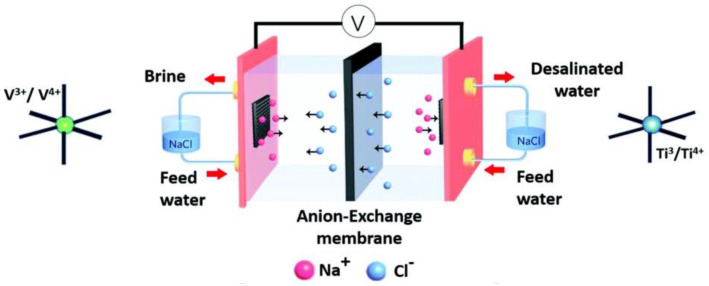
Illustration of the sodium ion desalination device. Reproduced with permission from [48]. Copyright 2020 Royal Society of Chemistry.

Sodium super ion conductor (NASICON) materials are a family of battery materials with outstanding stability [75] and excellent ion diffusion [76], which are beneficial to a good energy consumption performance. Generally, the NASICON materials are represented as AMM’(XO_4_)_3_ [77]. A is the alkali metal ion while M and M’ refer to the ions of transition metals. In the NASICON family, vanadium-based and titanium-based materials have mostly been studied. Cao et al. [78] employed an Na_3_V_2_(PO_4_)_3_/C cathode in an HCDI device with AC as the anode. Using the constant voltage mode, the cell reached an ultrahigh desalination performance of 137.20 mg·g^−1^ and a specific energy consumption of 0.464 Wh·g_NaCl_^−1^. NaTi_2_(PO_4_)_3_ is one of the few aqueous sodium-intercalation anodes and has appeared as an emerging CDI battery electrode in recent research. Guo et al. [79] coupled an NaTi_2_(PO_4_)_3_/C anode with an AC cathode. Using the CC mode, a low energy consumption of 0.112 Wh·g_NaCl_^−1^ was obtained at a current density of 10 mA·cm^−2^ with an initial salt concentration of 600 mM. Na_2_VTi(PO_4_)_3_, an NASICON material that has a structure similar to Na_3_V_2_(PO_4_)_3_ and the anode material NaTi_2_(PO_4_)_3_, exhibited an extra-low energy consumption of 0.068 Wh·g_NaCl_^−1^ at a current density of 0.075 A·g^−1^ in an MCDI system with an initial NaCl concentration of 1500 ppm as reported by Vafakhah et al. [48]. Na_2_VTi(PO_4_)_3_ could act as both the cathode and anode by releasing sodium ions when charged with a positive voltage or adsorbing sodium ions when charged with a negative voltage. In the system, which was divided by the anion-exchange membrane, when the cell was charged, the chloride ions in the salt solution were attracted through the membrane and the sodium ions were inserted into the anode, which is similar to the CID system. In this case, the cell is also denoted as a sodium ion desalination (NID) device, as illustrated in Figure 3 [48].

Metal oxides, which are regarded as precursors for ion intercalation, are classified into battery materials in this study. Liu et al. [80] constructed an HCDI cell with a sodium-intercalation electrode V_2_O_5_ and a capacitive electrode AC. The one-dimensional structure of V_2_O_5_ prepared from V_2_C MXene guaranteed smooth ion diffusion. The cell exhibited a low energy consumption of 0.16 Wh·g_NaCl_^−1^ with an initial salinity of 500 ppm at a current density of 0.03 A·g^−1^ (−0.4–0.8 V). Santos et al. [81] used γAl_2_O_3_/CNT as the cathode and TiO_2_/CNT as the anode in an asymmetric CDI cell. The CDI system exhibited a specific energy consumption of 0.18 Wh·g_NaCl_^−1^ with the CC charging mode at a current density of 0.0075 A·g^−1^ (0–1.2 V), which was 90.5% lower than that of a symmetrical CDI cell with AC electrodes.

In addition to the materials of sodium or chloride ion batteries, other battery materials have also been utilized as CDI electrodes lately. Lithium ion battery materials are widely used in our daily life, which are promising CDI electrodes as their massive production is favorable for scaled-up applications. Guo et al. [82] employed Li_4_Ti_5_O_12_/C as the anode in an HCDI system with carbon cloth as the counter electrode. The Li_4_Ti_5_O_12_/C anode showed sodium intercalation/deintercalation behaviors in an electrochemical test and was utilized as the sodium-insertion electrode in the desalination process. The cell showed a low energy consumption of 0.57 Wh·g_NaCl_^−1^ at a constant current density of 0.16 mA·cm^−2^, nearly half of the CV mode. Furthermore, MXenes are two-dimensional metal carbide or carbonitride materials suitable for intercalation of ions that are not just limited to sodium and chloride ions and have been studied as promising ion battery electrodes [83,84]. Ma et al. [85] used the MXene material Ti_3_C_2_T_x_ etched from Ti_3_AlC_2_ in a symmetric CDI cell. A good desalination capacity of 68 mg·g^−1^ and a low energy consumption of 0.24 Wh·g_NaCl_^−^^1^ were observed at a constant current of 0.02 A·g^−1^.

**Figure 4:**
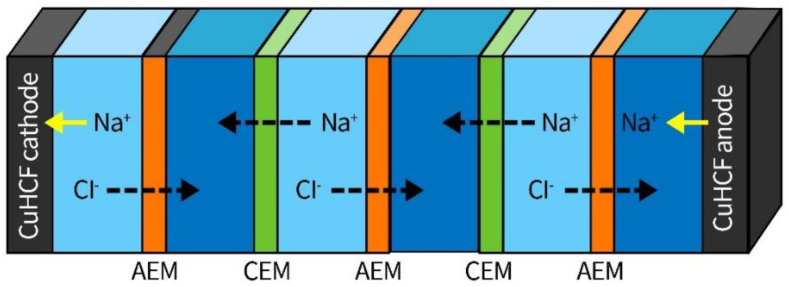
An MCDI cell with battery electrodes and five ion-exchange membrane stacks. Reproduced with permission from [86]. Copyright 2017 American Chemical Society.

Extremely low energy consumption can be achieved by combining intercalation materials with membrane stacks. As studied by Kim et al. [86], five ion-exchange membranes were introduced into a CDI cell with copper hexacyanoferrate (CuHCF) electrodes, as shown in Figure 4 [86], resulting in an enhanced desalination capacity; thus, energy consumption of 0.01 Wh·g_NaCl_^−1^ was observed.

Collectively, the use of battery materials is an effective way to achieve a good desalination capacity and energy consumption simultaneously. The low energy consumption of intercalation materials is mainly due to their higher charge efficiencies, and the details are listed in Table 1, where the information of some battery electrodes not mentioned in the main text is also included. 

### 4.3. Ion-Exchange Materials

The use of materials with ion-exchange functional groups as CDI electrodes is also a feasible choice to reduce energy consumption. The ion-exchange groups provide active sites for the adsorption of salt ions. The functional groups break the restrictions of pure EDL behaviors and alleviate the co-ion effect, which is a mechanism with some common ground with that of intercalation materials. However, the ion-exchange groups studied in CDI research are relatively limited.

Haq et al. [94] prepared polyamine/carbon composites with amino or sulfonic-acid groups as CDI electrodes, which exhibited a charge efficiency of 90% and desalination capacity of 17.7 mg·g^−1^, which was higher than those of original polyamine/carbon composites (86% and 14.7 mg·g^−1^), indicating less energy consumption. Wu et al. [95] constructed a CDI cell with two activated carbon electrodes prepared by spray-coating with a cation exchange dispersion and anion exchange, respectively. A low energy consumptions of 0.56–0.79 Wh·g_NaCl_^−1^ at a constant voltage of 1.2 V during the CDI cycles were obtained, which is far below that of conventional CDI with pure AC electrodes (1.57–0.85 Wh·g_NaCl_^−1^).

### 4.4. Materials with High Wettability

Materials with higher wettability have higher desalination capacities [96]; however, there is a lack of studies focusing on the effect of material wettability on CDI energy consumption. Recent studies have shown that the energy consumed during CDI can be reduced due to better ion diffusion enhanced by the higher wettability of the electrode material.

Yan et al. [97] prepared graphene/N-doped carbon composites as the CDI electrodes for desalination. Compared to pristine graphene, the contact angle of the composite decreased from 62° to 35°, signifying better wettability, which was due to the many defects that resulted from nitrogen doping. The graphene/N-doped carbon composites showed an energy consumption of 0.82 Wh·g_NaCl_^−1^ and a salt removal capacity of 14.5 mg·g^−1^ at a voltage of 1.2 V, which were 58.2% and 245.2% higher than those of the original graphene electrodes. Sodium iron hexacyanoferrate (NaFeHCF) was incorporated with acid-pretreated carbon nanotubes to obtain NaFeHCF/CNT composites by Zhang et al. [98]. The carboxyl groups of CNT produced by the acid provided composites with good wettability and the contact angle was reduced by 54.6% to 42.2° in comparison with pristine NaFeHCF. The NaFeHCF/CNT composites delivered an energy consumption that was 17.4% lower than that of NaFeHCF at an initial salinity of 3000 ppm under the constant voltage mode.

### 4.5. Ion-Adsorption Electrolytes in Redox Flow Desalination

**Figure 5:**
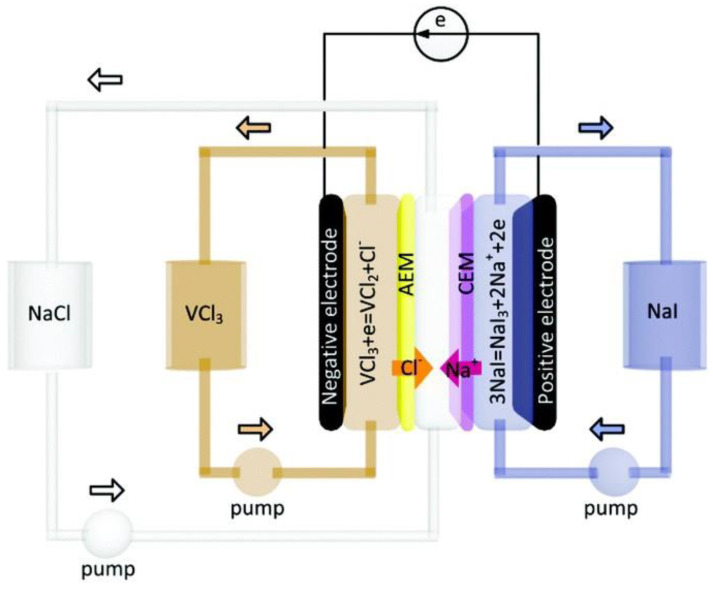
A typical redox flow desalination cell. Reproduced with permission from [99]. Copyright 2018 Royal Society of Chemistry.

The redox flow desalination cell is similar to the redox flow battery, in which liquid redox electrolytes rather than solid electrode materials are used as the main ion adsorption regions. As shown in Figure 5 [99], the redox desalination system is divided into three compartments by an anion-exchange membrane and a cation-exchange membrane: the cathodic region consists of the cathode and catholyte, the feed solution, and the anodic region consists of the anode and anolyte. However, in some cases, there are still some solid electrode materials on the current collectors. During charging, the solutes in the electrolytes are oxidized or reduced, leading to variations in the valences, thus attracting salt ions through the membranes due to charge compensation. Redox desalination often has a high charge efficiency due to the ion-exchange membranes and fast ion diffusion because of the short ion diffusion path; therefore, low energy consumption is expected [100].

Hou et al. [99] constructed a typical redox flow deionization cell with VCl_2_/VCl_3_ solution as the anolyte and NaI/NaI_3_ solution as the catholyte. The system showed an extra-low energy consumption of 0.49 Wh·g_NaCl_^−1^ with a constant current density of 0.22 mA·cm^−2^ within the voltage window of 0.3–1.1 V. Chen et al. [101] employed ferri/ferri cyanide solution as the catholyte and anolyte simultaneously based on the reversible redox couple Fe(CN)_6_^4−^/Fe(CN)_6_^3−^. Thus, an even lower energy consumption of 0.036 Wh·g_NaCl_^−1^ under the CC mode was achieved owing to a narrow voltage window of about 100 mV. Moreover, in addition to liquid solutions, the energy consumption can also be enhanced by optimization of the solid electrode materials. Ramalingam et al. [102] replaced graphite electrodes in a redox flow cell with the conductive polymer material 3,4-ethylene-dioxythiophene (PEDOT) with a rough surface. With a constant current density of 2 mA·cm^−2^, the energy consumption dramatically decreased by 75.34% to 0.18 Wh·g_NaCl_^−1^ and the salt removal rate improved by 38.74%.

## 5. The Energy Recovery of CDI Desalination

**Figure 6:**
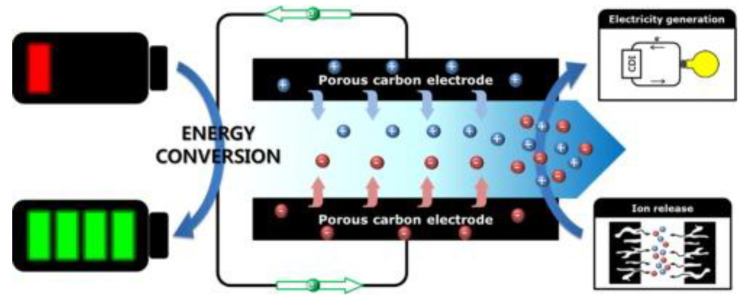
An example of energy recovery of CDI. Reproduced with permission from [103]. Copyright 2016 Elsevier.

CDI is the process of charging, and energy is stored during desalination. Energy can be regenerated during the discharging process, thus reducing the total energy consumption of CDI, as depicted in Figure 6 (CDI with carbon-based electrodes as the example) [103]. The energy recovery rate (ratio between the recovered and consumed energy during CDI) is a critical parameter used to assess the energy recovery performance. Recovery of the energy consumed during desalination is the future tendency of CDI energy savings.

### 5.1. Energy Recovery of CDI with Carbon Electrodes

Shiue et al. [66,104] used the energy regenerated from CDI to produce ozone, which is an early example of CDI energy recovery. Furthermore, a CDI desalination device with carbon electrodes coupled with energy storage was investigated by Pernia et al. [105] and a high ratio of up to 84% of the energy consumed was recovered with a frequency control discharging mode, which has rarely appeared in the literature. Moreover, the energy recovery can be enhanced by optimizing the circuit connection. In a study conducted by Andres et al. [106], the energy recovery rate of one single CDI cell with a constant desalination voltage of 1.2 V was as low as 11%; however, 70% of the consumed energy was regenerated by appropriate short-circuiting after the application of 6.0 V to 5 straight-connected CDI stacks during desalination. As lower energy consumption can be obtained with the introduction of an ion-exchange membrane, Dlugolecki et al. [107] tested the energy regeneration performances in MCDI. A high energy recovery of 84% was achieved with the traditional CC mode and the total energy consumption was 0.44 Wh·g_NaCl_^−1^, 74% lower than that of reverse osmosis. Moreover, a lower charge transfer resistance can result in a higher energy recovery. Rommerskirchen et al. [108] increased the feed concentration from 1000 to 60,000 ppm, and the energy recovery rate increased from about 12% to 36.2%.

Research on the CDI energy recovery of carbon-based electrodes at large scales has been carried out. Tan et al. [109] constructed an MCDI plant with carbon electrodes with a water productivity of 5 m^3^ per day, the operation mode of which was constant current, and the energy regeneration rate was 62%. A plant was further built on a similar scale but with higher water recovery (from 73.2% to 87%) [110]. By adding a bypass valve to adjust the flow mode, a total energy consumption of 0.57 Wh·g_NaCl_^−1^ was achieved with an energy recovery of nearly 40%.

### 5.2. Energy Recovery of CDI with Battery Electrodes

Lower total energy consumption can be obtained by recovering energy consumed in the CDI system with energy-efficient battery materials. Vafakhah et al. [72] used a Prussian blue electrode in an HCDI device with rGO as the counter electrode, in which a low total energy consumption of about 0.15 Wh·g_NaCl_^−1^ and an energy recovery ratio of 39% was acquired. A lower total energy consumption of 0.13 Wh·g_NaCl_^−1^ was found by Huang et al. [43]. Two battery electrodes were coupled together at the same feed salinity. However, the energy recovery rates of the above two studies were below 50%, indicating the promising potential of optimization to obtain even better total energy costs. Chen et al. [111] used the electrode pair of NaTi_2_(PO_4_)_3_/graphene and Ag, similar to the research carried out by Huang et al. [43], in CDI using the CC mode at a high salt concentration of 35,000 ppm. A high energy recovery rate of 71.9% was acquired together with a lower total energy consumption of 0.11 Wh·g_NaCl_^−1^.

Novel battery material couples have been developed to obtain good energy recovery performances. Guo et al. [112] synthesized an Ni, Co-bimetallic metal oxide framework/black phosphorus cathode for CDI with an Ag/rGO anode. The metal oxide framework (MOF) already had a high sodium-ion diffusion coefficient of 6.0 × 10^−12^ cm^2^·s^−1^, which was further increased 6.7 × 10^−12^ cm^2^·s^−1^ by the introduction of black phosphorus. In addition, the black phosphorus provided not only high electric conductivity but also negative charged functional groups that suppressed the co-ion effect. With the reduction of both the charge transfer and ion diffusion polarization, the CDI cell exhibited an extra-low total energy consumption of 0.034 Wh·g_NaCl_^−1^ and a high energy recovery ratio of 70.7%. Moreover, Wang et al. [113] coupled iron hexacyanoferrate with the π-conjugated conductive polymer polypyrrole/SO_4_^2^, and an even lower total energy consumption of 0.0089 Wh·g_NaCl_^−1^ was achieved with the energy recovery, which was approximately 6.7% of that of the RO technology [114].

### 5.3. Energy Recovery of Redox Flow Desalination

CDI employs capacitive or intercalation materials as electrodes, and tremendous progress has been achieved regardless of the desalination capacities or total energy consumption performance. However, polarization of solid materials can lead to problems such as electrode degradation, unsatisfactory ion diffusion kinetics, and the ion adsorption capacity being limited to the electrochemical capacity. These difficulties can be avoided if suitable substitutes for the solid electrodes are employed. As such, redox flow desalination is another promising choice that combines a high desalination capacity, good cycling stability, fast kinetics, and low total energy consumption. A redox flow battery can reach a lifespan of 10,000 to 20,000 cycles, indicating the potential steadiness in the redox flow desalination. Moreover, this technique is also suitable for handling water with high salt concentrations by utilizing the liquid ion adsorption regions, which removes the shackles of electric double layers and ion intercalations. Thus, energy recovery in redox flow desalination deserves special attention.

As mentioned previously, Hou et al. [99] carried out a classical redox flow desalination study, in which the energy recovery rate was 52.4% with a total energy consumption of 0.092 Wh·g_NaCl_^−1^. Desai et al. [115] used the redox couples of Zn/Zn^2+^ and Fe(CN)_6_^4−^/Fe(CN)_6_^3−^ to desalinate seawater and a lower total energy consumption of 0.070 Wh·g_NaCl_^−1^ was achieved. Khalla et al. [116] further replaced the Fe(CN)_6_^4−^/Fe(CN)_6_^3−^ couple with Br_3_^−^/Br^−^ in a redox flow desalination cell, in which the concentration of the feed solution was desalinated from about 30 g·L^−1^ to nearly 0 g·L^−1^ and the energy recovery was as high as 85% with a total energy consumption of 0.13 Wh·g_NaCl_^−1^.

Qian et al. [117] proposed an innovative concept of an “organic flow desalination battery”, which employed an organic redox electrolyte instead of inorganic counterparts and exhibited an energy recovery of 25%. The organic solutes in the catholyte and anolyte of the system were 4-hydroxy-2,2,6,6-tetramethylpiperidin-1-oxyl (TEMPO) and riboflavin-5′-phosphate sodium salt dehydrate (FMN-Na), respectively. Compared to a desalination cell with only aqueous electrolytes, it is possible for a high voltage to be applied for faster ion diffusion in this novel type of device owing to the larger voltage windows of the organic electrolytes. Kim et al. [118] fabricated an organic flow desalination system with 2-phenyl-4,4,5,5-tetramethylimidazoline-1-oxyl-3-oxide (PTIO) as both the catholyte and anolyte. The device was charged by 2.4 V during desalination and delivered a total energy consumption of 1.04 Wh·g_NaCl_^−1^ considering energy regeneration. The energy recovery of the organic flow desalination battery was upgraded to a competitive level by Debruler et al. [119] with the combination of both organic and inorganic solvents. The cell employed Na_4_Fe(CN)_6_ solution as the catholyte and methyl viologen as the anolyte, exhibiting an ultra-low total energy consumption of 0.070 Wh·g_NaCl_^−1^ with a high energy recovery of 79.7%.

### 5.4. The Effect of Operation Conditions on the Energy Recovery of CDI 

Apart from the appropriate selection of battery materials, optimization of the operation parameters is another practical choice to further enhance the energy recovery of CDI.

According to the present study, an increased electric intensity is not favorable for energy recovery. In the study conducted by Chen et al. [111], with an increase in the current density from 1000 to 6000 mA·g^−1^, the energy recovery ratio decreased from 71.9% to 65.0%. Additionally, the energy recovery rate drastically decreased from 51% to 10% by changing the current density from 20 to 5 A·m^−2^ in a flow-through CDI cell with CuHCF electrodes as reported in a study by Son et al. [120], in which the flow-through mode interestingly had a lower energy efficiency during desalination but a higher energy recovery than that of the flow-by mode.

Higher salinity can enhance energy recovery by offering a higher solution conductivity. Chen et al. [121] increased the salt concentration in the CDI module from 5 to 50 mM, and the energy recovery rate significantly increased from 34.8% to 49.6%. Lee et al. [122] placed side salt solution between the feed solution and battery electrodes with ion-exchange membranes as the separators of the solutions. The energy recovery increased while the side solution concentration increased at a lower electric resistance and the system reached an energy recovery rate of 73%. Moreover, Nam et al. [123] improved the energy recovery by changing the PH value of the electrolyte in the regeneration process. A high energy recovery ratio of 75.6% was obtained in a hydrochloric acid solution with a pH of 1.2, over twice of that in a neutral solution.

The details of the energy recovery performances are presented in Table 2. The energy recovery rate can surpass 80% while the total energy consumption can be reduced to below 0.01 Wh·g_NaCl_^−1^, indicating the robust potential of energy recovery of CDI. It is also obvious that the energy recovery performance is influenced by numerous factors. Only by comprehensively considering such factors can satisfactory optimization of the energy recovery in CDI be achieved.

## 6. Conclusions

Various factors were reviewed in this study, and favorable elements for lower energy consumption were demonstrated. Regarding the external conditions of CDI, a constant current charging mode, weaker electric field, and lower flow rate are all beneficial for a better energy consumption performance but relatively worse desalination capacity. However, an initial feed concentration that is too low can cause high energy consumption due to a low charge efficiency, which is an exception among the external factors.

On the other hand, internal factors include ion-exchange membrane incorporation, flow direction, and electrode material/electrolyte, which are all critical elements. With the use of an ion-exchange membrane or a flow-by configuration, lower energy consumption is likely to be obtained. Moreover, electrode materials with ion intercalation/deintercalation behaviors, ion-exchange functional groups, or high wettability are preferred for achieving better energy efficiencies. The intercalation/deintercalation or ion-exchange reactions provide a semi-selective surface to alleviate the co-ion effect and enhance the charge efficiency while higher wettability might lead to better ion diffusion. In addition, flow redox desalination with redox electrolytes appears to be quite promising due to the extra-low energy consumption and the potential of a long lifespan of up to 20,000 cycles. The redox electrolyte can break the ion storage limits of the solid-liquid surface of the electrode material, provide a smooth diffusion of ions, and avoid structural instability of the solid electrode simultaneously.

Furthermore, the energy recovery of CDI is another robust choice for reducing energy consumption. Based on the state-of-the-art literature, over 80% of the consumed energy can be regenerated in CDI, indicating a feasible routine for further decreasing energy consumption. Among the major industrial desalination technologies being applied worldwide, reverse osmosis is assumed to be the most energy efficient. When desalting a salty feed solution of around 30,000 ppm, the energy cost of RO with energy recovery is nearly 13.98 times higher than that of CDI, signifying the advantageous energy efficiency of CDI.

Energy consumption is influenced by a number of factors. Therefore, the consumed energy in CDI can be optimized by integrating the preferred elements, among which it is assumed that the coupling of flow redox desalination and energy recovery is an option with good potential. The combination offers a high energy efficiency, high charge efficiency, high desalination performance, and long cycle life in CDI research, signifying a prospective direction of the desalination field. Moreover, redox flow desalination with energy recovery combines the advantages of extra-low total energy consumption (<0.1 Wh·g_NaCl_^−1^) and an extra-long life cycle (~20,000 cycles), indicating a low operation cost and high investment reward, respectively. Therefore, promising and exciting future directions of the desalination industry are revealed.

## Figures and Tables

**Table 1:** Energy consumptions of battery materials of CDI in the literature.

Material Couple	Charging Mode	Voltage/Current Density and Voltage Window	Energy Consumption	Initial Salinity	Desalination Capacity	Charge Efficiency	Reference
Ag || NaTi_2_(PO_4_)_3_/rGO	CC	100 mA·g^−1^ (0~1.4 V)	about 0.18 Wh·g_NaCl_^−1^	2500 ppm	35.8 mg·g^−1^	---	[43]
Na_3_(VO)_2_(PO_4_)_2_/rGO || AC	CC	25 mA·g^−1^ (−1.4~1.4 V)	0.35 Wh·g_NaCl_^−1^	1000 ppm	175.94 mg·g^−1^	---	[49]
NiCHF || NiCHF	CC	1 mA·cm^−2^	0.033 Wh·g_NaCl_^−1^	100 mM	54.5–60 mg·g^−1^	about 86%	[50]
Fe_4_[Fe(CN)_6_]_3_/rGO || rGO	CC	100 mA·g^−1^ (−0.2~1.4 V)	0.23 Wh·g_NaCl_^−1^	2500 ppm	80 mg·g^−1^	---	[72]
BiOCl/C || Bi/C	CC	---	0.23 Wh·g_NaCl_^−1^	20 mM	---	---	[73]
AgCl || Ag	CC	100 mA·g^−1^ (−0.1~0.1 V)	0.059 Wh·g_NaCl_^−1^	600 mM	115 mg·g^−1^	98%	[74]
Na_3_V_2_(PO_4_)_3_/C || AC	CV	1.0 V	0.46 Wh·g_NaCl_^−1^	100 mM	137.20 mg·g^−1^	98.7%	[78]
AC || NaTi_2_(PO_4_)_3_/C	CC	10 mA·cm^−2^ (0~2.0 V)	0.11 Wh·g_NaCl_^−1^	600 mM	---	---	[79]
Na_2_VTi(PO_4_)_3_ || Na_2_VTi(PO_4_)_3_	CC	75 mA·g^−1^ (−0.1~0.1 V)	0.068 Wh·g_NaCl_^−1^	1000 ppm	90 mg·g^−1^	---	[48]
V_2_O_5_ || AC	CC	30 mA·g^−1^ (−0.4~0.8 V)	0.16 Wh·g_NaCl_^−1^	500 ppm	22.3 mg·g^−1^	---	[80]
γAl_2_O_3_/CNT || TiO_2_/CNT	CC	7.5 mA·g^−1^ (0~1.2 V)	0.18 Wh·g_NaCl_^−1^	10 mM	12.7 mg·g^−1^	85%	[81]
Carbon cloth || Li_4_Ti_5_O_12_/C	CC	0.16 mA·cm^−2^ (−1.4~1.4 V)	0.57 Wh·g_NaCl_^−1^	2500 ppm	25 mg·g^−1^	83%	[82]
MXene Ti_3_C_2_T_x_ || MXene Ti_3_C_2_T_x_	CC	20 mA·g^−1^ (−1.2~1.2 V)	0.24 Wh·g_NaCl_^−1^	585 ppm	68 mg·g^−1^	---	[85]
CuCHF || CuCHF	CC	1.4 A·m^−2^ (−0.6~0.6 V)	0.01 Wh·g_NaCl_^−1^	25 mM	---	---	[86]
Na_2_NiFe(CN)_6_|| NaNiFe(CN)_6_	CC	1.4 A·m^−2^ (−1.5~1.5V)	0.26 Wh·g_NaCl_^−1^	20 mM	about 27 mg·g^−1^	95%	[87]
MoS_2_ || Zn	CC	1.4 mA·cm^−2^ (0~3 V)	1.57 Wh·g_NaCl_^−1^	600 mM	1300 mg·g^−1^	70%	[88]
TiS_2_ || Carbon textile	CC	100 mA·g^−1^ (0~1.2 V)	0.68 Wh·g_NaCl_^−1^	600 mM	14.5 mg·g^−1^	>85%	[89]
Ti_3_C_2_T_x_ || Ti_3_C_2_T_x_/Ag	CC	50 mA·g^−1^ (about −1.2–1.2 V)	0.26 Wh·g_NaCl_^−1^	10 mM	128.40 mg·g^−1^	---	[90]
FePO_4_/rGO || rGO	CV	1.8 V	0.9 Wh·g_NaCl_^−1^	40 mM	85.94 mg·g^−1^	91.4%	[91]
Na_0.55_Mn_2_O_4_/Na_0.7_MnO_2_ || Na_0.55_Mn_2_O_4_/Na_0.7_MnO_2_	CV	1.0 V	0.55 Wh·g_NaCl_^−1^	50 mM	68.5 mg·g^−1^	84%	[92]
Sb || Porous carbon	CC	200 mA·g^−1^ (−2.0~2.0 V)	0.67 Wh·g_NaCl_^−1^	600 mM	748 mg·g^−1^	74%	[93]

**Table 2:** The energy recovery performances of different CDI electrodes.

Material/Electrolyte Couple	Initial Salinity	Energy Recovery Mode	Regeneration Electric Intensity	Energy Recovery Rate	Energy Consumption with Energy Recovery	Reference
Carbon || Carbon	---	frequency control	---	84%	---	[105]
Activated charcoal || Activated charcoal	5.5 mS·cm^−1^	short-circuiting	---	70%	---	[106]
Porous carbon || Porous carbon	273 mM	CC	1.69 A·m^−2^	about 84%	0.44 Wh·g_NaCl_^−1^	[107]
AC || AC (flow electrode)	60,000 ppm	CC	2.48 mA·cm^−2^	36.2%	0.44 Wh·g_NaCl_^−1^	[108]
Carbon || Carbon	4000 ppm	CC	---	62%	---	[109]
Carbon || Carbon	1900 ppm	CC	---	about 40%	0.57 Wh·g_NaCl_^−1^	[110]
AC || AC	50 mM	short-circuiting	---	49.6%	---	[121]
Fe_4_[Fe(CN)_6_]_3_/rGO || rGO	2500 ppm	CC	100 mA·g^−1^	39%	about 0.15 Wh·g_NaCl_^−1^	[72]
Ag/rGO || NaTi_2_(PO_4_)_3_/rGO	2500 ppm	CC	100 mA·g^−1^	over 30%	0.13 Wh·g_NaCl_^−1^	[43]
Ag/CNT || NaTi_2_(PO_4_)_3_/graphene	35,000 ppm	CC	1000 mA·g^−1^	71.9%	0.11 Wh·g_NaCl_^−1^	[111]
MXene Ti_3_C_2_T_x_ || MXene Ti_3_C_2_T_x_	10 mM	CC	20 mA·g^−1^	5.44%	0.23 Wh·g_NaCl_^−1^	[85]
Ni, Co MOF/black phorsphorus || Ag/rGO	synthetic seawater	CC	300 mA·g^−1^	70.7%	0.034 Wh·g_NaCl_^−1^	[112]
Iron hexacyanoferrate || Polypyrrole/SO_4_^2−^	30,339 ppm	CC	1.88 mA·cm^−2^	65%	0.0089 Wh·g_NaCl_^−1^	[113]
CuCHF || CuCHF	50 mM	CC	5 A·m^−2^	51%	0.017 Wh·g_NaCl_^−1^	[120]
NiCHF || Ag	50 mM	CC	5 A·m^−2^	73%	---	[122]
CuCHF || Bi	0.6 M	CC	1 mA·cm^−2^	75.6%	---	[123]
NaI/NaI_3_ || VCl_2_/VCl_3_ (redox flow)	about 19,000 ppm	CC	0.22 mA·cm^−2^	52.4%	0.092 Wh·g_NaCl_^−1^	[99]
K_4_Fe(CN)_6_/K_3_Fe(CN)_6_ || ZnCl_2_ (redox flow)	35,000 ppm	CC	2.48 mA·cm^−2^	over 80%	0.070 Wh·g_NaCl_^−1^	[115]
Br_2_/NaBr || ZnCl_2_ (redox flow)	29,220 ppm	CC	2 mA·cm^−2^	85%	0.13 Wh·g_NaCl_^−1^	[116]
TEMPO || FMN-Na (redox flow)	1 M	CC	0.13 mA·cm^−2^	25%	---	[117]
PTIO || PTIO (redox flow)	50 mM	CC	5 mA·cm^−2^	---	1.04 Wh·g_NaCl_^−1^	[118]
Na_4_Fe(CN)_6_ || methyl viologen (redox flow)	560 mM	CC	1.33 mA·cm^−2^	79.7%	0.070 Wh·g_NaCl_^−1^	[119]

## Supplementary Materials

The following supporting information can be downloaded at: https://www.mdpi.com/article/10.3390/ijerph191710599/s1. References in supplementary file are [29,40,41,42,124,125,126,127].
